# Predictors of Treatment Outcome in Persons With Anorexia Nervosa: On the Practice of Regressing Body Mass Index at the End of Treatment on Body Mass Index at Baseline

**DOI:** 10.1002/eat.24324

**Published:** 2024-11-12

**Authors:** Adrian Meule, David R. Kolar, Ulrich Voderholzer

**Affiliations:** ^1^ Department of Psychology University of Regensburg Regensburg Germany; ^2^ Schoen Clinic Roseneck Prien am Chiemsee Germany; ^3^ Department of Psychiatry and Psychotherapy LMU University Hospital Munich Germany; ^4^ Department of Psychiatry and Psychotherapy University Hospital of Freiburg Freiburg Germany

**Keywords:** anorexia nervosa, body mass index, outcome, predictors, therapy, treatment, weight gain

## Abstract

**Objective:**

It is often stated that a higher body mass index (BMI) at the beginning of treatment predicts a better weight outcome at the end of treatment in persons with anorexia nervosa (AN). However, this interpretation is based on the between‐persons relationship of BMI at the two measurements, which primarily reflects the fact that the rank‐ordering of persons according to their BMI is quite stable over time. In contrast, a lower BMI at baseline is related to a larger BMI change, which primarily reflects the fact that the variance of BMI at the end of treatment is larger than that at baseline. This study aimed to demonstrate these relationships empirically and caution against interpreting BMI at baseline as a predictor of BMI at discharge or BMI change.

**Method:**

Changes of BMI from admission to discharge were analyzed based on 4863 persons with AN (97% female) who received inpatient treatment between 2015 and 2024.

**Results:**

BMI at admission was positively related to BMI at discharge (*r* = 0.55) but negatively related to BMI change from admission to discharge (*r* = −0.39).

**Discussion:**

While it is true that a higher BMI at baseline is associated with a higher BMI at the end of treatment, a lower BMI at baseline is actually related to a larger weight gain during treatment. Yet, concluding that the treatment is more effective for patients with low or high BMI at baseline would be incorrect in either case, as the independent and dependent variables are the same variables measured at different time points.

**Summary**

- Body mass index (BMI) at the beginning of treatment positively correlates with BMI at the end of treatment in persons with anorexia nervosa.
- BMI at the beginning of treatment negatively correlates with BMI change during treatment.
- This means that a patient with a low body weight at the beginning of treatment will likely gain more weight—but will likely still have a lower body weight at the end of treatment—than a patient with a high body weight at the beginning of treatment.
- This does not necessarily mean that either of these patients responds better or worse to the treatment, as this more likely reflects the large between‐persons correlation between BMI at the two measurements and the to‐be‐expected inverse correlation between baseline and change scores.

## 1 Introduction

Anorexia nervosa (AN) is an eating disorder marked by significantly low body weight, which is accompanied by behaviors aimed at reducing energy intake (e.g., restrictive eating, purging) or increasing energy expenditure (e.g., excessive exercise), typically associated with a fear of weight gain. As low body weight is the core symptom of AN, a lower body mass index (BMI, kg/m^2^) indicates higher severity in both DSM–5 (American Psychiatric Association 2013) and ICD–11 (World Health Organization 2022). Accordingly, weight restoration usually is the primary goal in AN treatment (Marzola et al. 2013; Voderholzer et al. 2020), along with changing eating disorder‐related cognitions and behaviors.

When examining predictors of weight outcomes in persons with AN, BMI at the beginning of treatment is often cited as one of the most robustly found predictor variables such that a higher BMI at the beginning of treatment is related to a better treatment outcome and a lower BMI at the beginning of treatment is related to a poorer treatment outcome (Gorrell, Hail, and Reilly 2023). For example, it has been reported that premorbid BMI “predicts” BMI at the beginning of treatment, which in turn “predicts” BMI at the end of treatment, which in turn “predicts” BMI at 1‐year follow up (Föcker et al. 2015). A problem with this interpretation is that when the same variable is measured at two time points and the later time point is regressed on the earlier time point, such an analysis does not test any changes over time. In a simple linear, ordinary‐least‐squares regression model—that is, a model that only includes one independent variable—the coefficient for the relationship between the independent variable (e.g., BMI at admission) and the dependent variable (e.g., BMI at discharge) merely quantifies the between‐persons relationship between the two variables. In fact, the standardized regression coefficient in such a model is equal to Pearson's correlation coefficient (Darlington and Hayes 2017, p. 31). Thus, a positive and statistically significant regression coefficient in a model that includes BMI at admission as an independent variable and BMI at discharge as a dependent variable indicates that the rank‐ordering of persons according to their BMI stays quite stable from admission to discharge: a person that has a higher BMI than another person at admission is likely to have a higher BMI at discharge than this person at discharge as well.

For examining predictors of changes over time, baseline values of the dependent variable need to be controlled for. However, this is not possible here, as BMI at the beginning of treatment is already included in this model as the sole independent variable. One possibility to examine whether BMI at the beginning of treatment predicts weight change would be to construct a difference score (e.g., by subtracting BMI at admission from BMI at discharge) and then regress this BMI change on BMI at the beginning of treatment. Doing so will likely result in a negative coefficient for the effect of BMI at admission on BMI change, which is determined by the correlation between and the variance of the two measurements (Clifton and Clifton 2019). That is, there will always be a correlation between the change score and the baseline values, regardless of any treatment effects (Clifton and Clifton 2019).

The aim of this study was to demonstrate these relationships empirically by analyzing data from a large sample of persons with AN who received inpatient treatment. We then discuss what these relationships actually represent and how they should be interpreted.

## 2 Methods

### 2.1 Samples

Data of 4863 persons with AN (ICD–10 code F50.0) who received inpatient treatment (details of which are described elsewhere; e.g., Meule and Voderholzer 2022) at the Schoen Clinic Roseneck (Prien am Chiemsee, Germany) between 2015 and 2024 were analyzed. Sample characteristics are displayed in Table 1. According to the guidelines by the ethics committee of the LMU Munich, retrospective studies conducted on already available, anonymized data are exempt from requiring ethics approval.

**TABLE 1:** Descriptive statistics of study variables and correlations with BMI at admission and BMI change.

*N* = 4863	Descriptive statistics	*r*_BMI admission_	*r*_BMI change_
BMI (kg/m^2^) at admission	*M* = 15.0 (*SD* = 1.9)	—	−0.387 (*p* < 0.001)
BMI (kg/m^2^) at discharge	*M* = 17.5 (*SD* = 2.1)	0.552 (*p* < 0.001)	0.555 (*p* < 0.001)
BMI (kg/m^2^) change	*M* = 2.5 (*SD* = 1.9)	−0.387 (*p* < 0.001)	—
Age (years)	*M* = 22.3 (*SD* = 9.8)	−0.075 (*p* < 0.001)	−0.004 (*p* = 0.788)
Sex (0 = female, 1 = male)	3.3% (*n* = 160)	0.088 (*p* < 0.001)	−0.034 (*p* = 0.018)
Length of stay (days)	*M* = 92.5 (*SD* = 55.4)	−0.182 (*p* < 0.001)	0.679 (*p* < 0.001)
Any comorbid mental disorders (0 = no, 1 = yes)	65.2% (*n* = 3170)	0.068 (*p* < 0.001)	−0.015 (*p* = 0.298)

*Notes: r* indicates Pearson's correlation coefficients when both variables are continuous and point‐biserial correlation coefficients when one of the variables is dichotomous.

### 2.2 Data Analyses

Data were analyzed with R version 4.3.2 in RStudio version 2023.12.1. Note that it is generally recommended to use age‐ and sex‐adjusted BMI for children and adolescents because of growth‐related changes in body composition (Cole et al. 2000). However, BMI‐SDS (obtained with the R package childsds using German reference data) was highly correlated with BMI in the current sample (*r* = 0.85), which is why we only analyzed BMI to avoid redundancy. Changes in BMI from admission to discharge were tested with a paired samples *t*‐test. The relationships of study variables with BMI at admission and BMI change (BMI at admission subtracted from BMI at discharge) were tested with Pearson's correlation coefficients and point‐biserial correlation coefficients. To demonstrate that the standardized coefficient in a linear, ordinary‐least‐squares regression model is equivalent to Pearson's *r* when there is only one independent variable, BMI at discharge and BMI change were regressed on BMI at admission in two separate models. To further visualize BMI change as a function of BMI at admission, we arbitrarily created a group of persons with a BMI ≤ 15 kg/m^2^ (*n* = 2361) and a group of persons with a BMI > 15 kg/m^2^ (*n* = 2502) and formally tested differential changes in BMI from admission to discharge as a function of these groups with an analysis of variance for repeated measures. The data and code with which the results reported here can be reproduced and accessed at https://osf.io/9v3fk.

## 3 Results

**FIGURE 1:**
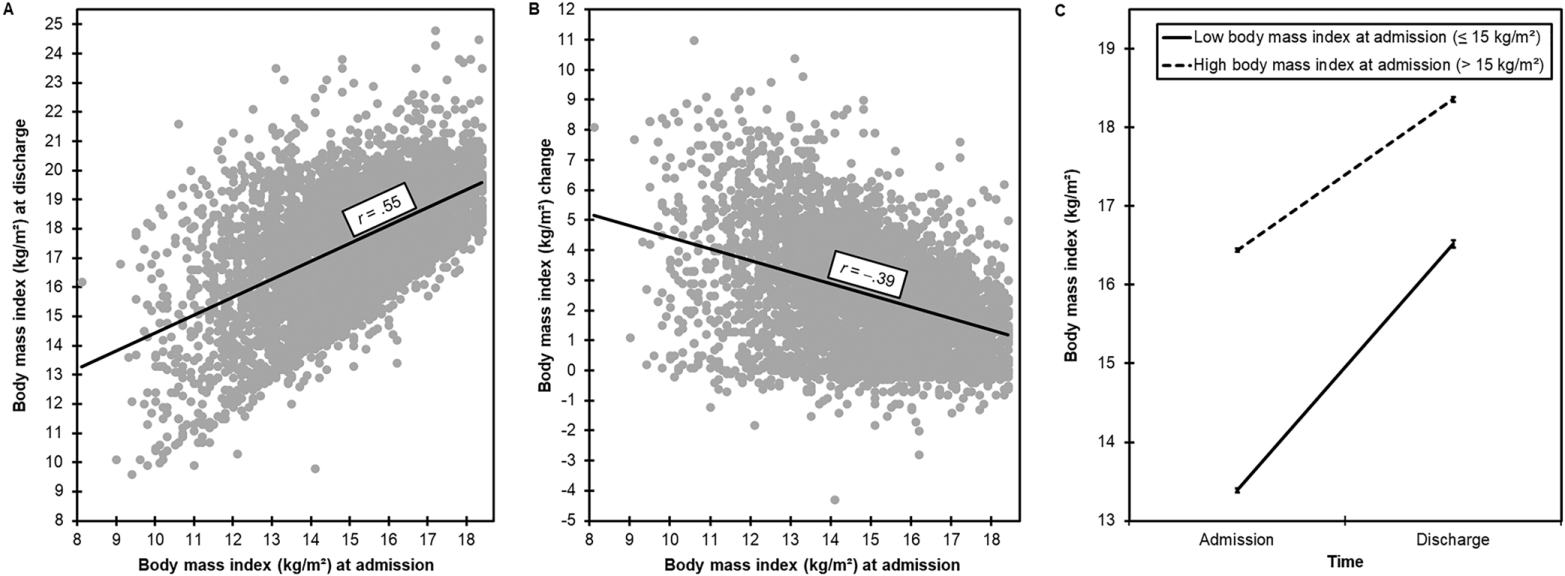
Panel A displays a scatterplot with a linear trend line, visualizing the relationship between BMI at admission and discharge. Panel B displays a scatterplot with a linear trend line, visualizing the relationship between BMIat admission and the difference score of BMI at admission subtracted from BMI at discharge. Panel C displays mean BMI at admission and discharge as a function of persons with a BMI ≤ 15 and > 15 kg/m^2^ at admission. Error bars represent standard errors of the mean. Note that these analyses were based on arbitrarily categorizing the sample into these two groups, but this depiction only serves the purpose of visualizing the effect displayed in Panel B in a different way.

BMI increased from admission to discharge with a large effect size (*t*_(4862)_ = 93.2, *p* < 0.001, *d* = 1.3; Table 1). BMI at admission positively correlated with BMI at discharge with a large effect size (Figure 1A) and negatively correlated with BMI change with a medium effect size (Figure 1B and Table 1). As outlined in the Introduction section, the correlation coefficients (Figure 1 and Table 1) are equal to the standardized coefficients when regressing BMI at discharge on BMI at admission (*β* = 0.55, *p* < 0.001) and when regressing BMI change on BMI at admission (*β* = −0.39, *p* < 0.001). The effect of BMI at admission on BMI change was still negative and significant (*β* = −0.27, *p* < 0.001) when controlling for age, sex, length of stay, and comorbid mental disorders.

The interaction effect groups × time was significant (*F*_(1,4861)_ = 569.3, *p* < 0.001, *η* ^2^_*G*_ = 0.04), indicating that BMI change from admission to discharge differed in the two groups. Figure 1C visualizes the effect of BMI at admission on BMI change by displaying BMI at admission and discharge in persons with a BMI at admission ≤ 15 kg/m^2^ and > 15 kg/m^2^. As this depiction is merely a different way of displaying the effect presented in Figure 1B, persons with a lower BMI at admission showed a steeper slope (i.e., larger weight gain from admission to discharge) than persons with a higher BMI at admission. Despite this larger weight gain, BMI at discharge was still lower than in persons with a higher BMI at admission (Figure 1C).

## 4 Discussion

The current study demonstrates that the statement that a higher BMI at the beginning of treatment is a predictor of better weight outcomes in persons with AN is only partially true. BMI at admission correlates positively with BMI at discharge, indicating that a person who has a higher BMI than another person at admission also tends to have a higher BMI than this person at discharge. That is, regressing BMI at the end of treatment on BMI at the beginning of treatment does not answer the question whether a person with a low or high body weight at admission responds better or worse to the treatment. Instead, the positive coefficient rather indicates that interindividual differences in body weight remain quite stable across different time points.

When examining weight change, a lower BMI at admission is related to a larger weight gain. Importantly, this relationship is to be expected, as there is always a correlation between change and baseline scores in treatment studies—regardless of any treatment effect—which depends on the correlation between the two measurements and their variances, as described in formula #9 in the article by Clifton and Clifton (2019)

$$\text{corr}\left(Y-X,X\right)=\frac{{\rho \sigma }_{x}\sigma_{y}-\sigma_{x}^{2}}{\sigma_{x}\sqrt{\left(\sigma_{y}^{2}-2\rho \sigma_{x}\sigma_{y}+\sigma_{y}^{2}\right)}}=\frac{\rho \sigma_{y}-\sigma_{x}}{\sqrt{\left(\sigma_{y}^{2}-2\rho \sigma_{x}\sigma_{y}+\sigma_{y}^{2}\right)}}$$

In the current example, *X* would be BMI at admission, *Y* would be BMI at discharge, *σ* ^2^_*X*_ and *σ* ^2^_*Y*_ are their variances, respectively, and *ρ* is the correlation between *X* and *Y*. Entering the correlation between BMI at admission and BMI at discharge (0.55) for *ρ*, the standard deviation of BMI at admission (1.9) for *σ* ^2^_*X*_, and the standard deviation of BMI at discharge (2.1) for *σ* ^2^_*Y*_ in this formula yields a correlation of 0.39, that is, the correlation coefficient for the relationship between BMI at admission and BMI change (Table 1).

There are numerous factors that might influence this relationship. Statistically, difference scores tend to be negatively correlated with the initial state due to regression to the mean, that is, extreme values usually tend to get more moderate over time (i.e., extremely high values tend to decrease and extremely low values tend to increase). Correspondingly, the current findings mirror effects observed in the treatment of obesity: persons with a higher body weight at the beginning of treatment tend to lose more weight than those with a lower body weight (but despite this larger weight loss, those starting with a higher weight still have a higher weight at the end of treatment than those starting with a lower weight; Christiansen et al. 2007). Biologically, a larger weight gain in those with a lower BMI may be due to a lower resting metabolic rate at the beginning of treatment. Psychologically, many patients accept that they have to gain weight during treatment but refuse to exceed certain self‐imposed thresholds (e.g., 50 kg), which are reached faster by those who start with a higher BMI at admission. In line with all three of these factors, rates of weight gain usually level off during the course of treatment (Meule, Kolar, and Voderholzer 2022). In either case, an incorrect interpretation would be that the treatment is more effective for patients with a lower body weight at admission, as examining the correlation between BMI at admission and BMI change cannot answer this question.

Interpretation of the current findings is, of course, limited to inpatients with AN in Germany and may not translate to other treatment forms (e.g., day‐patient or outpatient treatment) or to countries with different healthcare systems. Moreover, the current analyses were restricted to BMI for the sake of brevity and clarity, but treatment outcome should be evaluated by not only using body weight but also considering other eating disorder symptomatology. Moreover, we only examined BMI at admission and discharge in the current study but would argue that results similarly apply to measurements before and after treatment. For example, the study by Föcker et al. (2015) showed that BMI was correlated across different measurements before, during, and after treatment. Similarly, a recent study reported that a higher premorbid BMI was related to a larger weight loss before treatment in persons with AN, again demonstrating the inverse correlation between baseline values and change scores (Hebebrand et al. 2024).

For clinicians, the current findings indicate that they can expect that a patient with a low body weight at the beginning of treatment will likely gain more weight—but will likely still have a lower body weight at the end of treatment—than a patient with a high body weight at the beginning of treatment. However, this does not necessarily mean that either of these patients responds better or worse to the treatment, as this more likely reflects the large between‐persons correlation between BMI at the two measurements and the to‐be‐expected inverse correlation between baseline and change scores. For the latter, statistical solutions have been proposed with cautionary notes (Tu and Gilthorpe 2007). If researchers are interested in examining the predictive effect of baseline BMI on BMI changes, we would recommend assessing BMI at multiple occasions, which would allow applying growth curve modeling (Curran, Obeidat, and Losardo 2010), in which between‐ and within‐persons effects as well as fixed and random effects can be separated more clearly than when using correlational analyses based on two measurements.

## Author Contributions

**Adrian Meule:** conceptualization, data curation, formal analysis, methodology, visualization, writing – original draft. **David R. Kolar:** supervision, writing – review and editing. **Ulrich Voderholzer:** resources, writing – review and editing.

## Conflicts of Interest

The authors declare no conflicts of interest.

### Open Research Badges

This article has earned an Open Data badge for making publicly available the digitally‐shareable data necessary to reproduce the reported results. The data is available at: https://osf.io/9v3fk.

## Data Availability

The data and code with which the results reported here can be reproduced can be accessed at https://osf.io/9v3fk.
